# Breast Cancer Polygenic Risk Score Validation and Effects of Variable Imputation

**DOI:** 10.3390/cancers16081578

**Published:** 2024-04-20

**Authors:** Jeffrey J. Beck, John L. Slunecka, Brandon N. Johnson, Austin J. Van Asselt, Casey T. Finnicum, Cheryl Ageton, Amy Krie, Heidi Nickles, Kenneth Cowan, Jessica Maxwell, Dorret I. Boomsma, Eco de Geus, Erik A. Ehli, Jouke-Jan Hottenga

**Affiliations:** 1 Avera Genetics, Avera McKennan Hospital & University Health Center, Sioux Falls, SD 57105, USAerik.ehli@avera.org (E.A.E.); 2 Avera Cancer Institute, Sioux Falls, SD 57105, USA; 3 Fred and Pamela Buffet Cancer Center and Eppley Institute for Research in Cancer at University of Nebraska Medical Center, Omaha, NE 68105, USA; 4 Department of Biological Psychology, Netherlands Twin Register, Vrije Universiteit Amsterdam, 1081 BT Amsterdam, The Netherlandsj.j.hottenga@vu.nl (J.-J.H.)

**Keywords:** breast cancer, polygenic risk score, validation, genetic risk, GWAS, genotype imputation

## Abstract

**Simple Summary:**

Breast cancer is the most common cancer in women and has been associated with genetic and environmental factors. New developments have led to the creation of genetic risk scores which seek to better approximate an individual’s risk of developing cancer and to be used clinically to improve cancer screening. Previous studies have shown that these risk scores can be used to determine an individual’s cancer risk, but replication in independent groups is limited. In addition, certain genotyping methods utilize a process called imputation, which has the potential to make polygenic risk scores less accurate. This work aims to validate two breast cancer polygenic risk scores and to interrogate the impact imputation has on their values to improve their clinical utility.

**Abstract:**

Breast cancer (BC) is a complex disease affecting one in eight women in the USA. Advances in population genomics have led to the development of polygenic risk scores (PRSs) with the potential to augment current risk models, but replication is often limited. We evaluated 2 robust PRSs with 313 and 3820 SNPs and the effects of multiple genotype imputation replications in BC cases and control populations. Biological samples from BC cases and cancer-free controls were drawn from three European ancestry cohorts. Genotyping on the Illumina Global Screening Array was followed by stringent quality control measures and 20 genotype imputation replications. A total of 468 unrelated cases and 4337 controls were scored, revealing significant differences in mean PRS percentiles between cases and controls (*p* < 0.001) for both SNP sets (313-SNP PRS: 52.81 and 48.07; 3820-SNP PRS: 55.45 and 49.81), with receiver operating characteristic curve analysis showing area under the curve values of 0.596 and 0.603 for the 313-SNP and 3820-SNP PRS, respectively. PRS fluctuations (from ~2–3% up to 9%) emerged across imputation iterations. Our study robustly reaffirms the predictive capacity of PRSs for BC by replicating their performance in an independent BC population and showcases the need to average imputed scores for reliable outcomes.

## 1. Introduction

Breast cancer (BC) is one of the most common cancers in the world, with 2.26 million new cases, and killing over 684,000 women in 2020 [1]. The disease has seen great reductions in recent decades [2] due to the increasing number of medical therapies, use of screening methods, and emphasis on preventative efforts [3,4]. Already, in both the United States and the Netherlands, BC screening begins at age 50 for average risk women, which has led to important decreases in BC-related mortality in both countries [3,5]. However, reductions in cancer incidence have slowed in multiple domains, especially those which have improved outcomes from early detection, such as BC [6]. Increasing preventative efforts has effectively decreased both the morbidity and mortality of cancers; however, addressing the underlying risk factors which lead to BC is still needed [7].

Enhancing the methods of BC prevention would play a significant role in reducing the number of women who will develop cancer in their lifetimes. One of the major risk factors in the development of BC is an individual’s genetic code. Many advances have been made to characterize the genetic underpinnings of BC [8], most notably the discovery of BRCA1 [9], CHEK2 [10], and ATM mutations [11]. While the identification of these genes has played a major role in understanding those at very high risk of familial BC, most women who develop BC do not possess these mutations [8]. In fact, approximately 0.12% of Caucasian women actually carry susceptible variants in BRCA1, accounting for only 1.7% of BC cases diagnosed before age 70 [9]. Heritability estimates of BC are between 15% and 31% [12,13,14], leaving considerable potential for other genetic variants to contribute to overall genetic risk for BC.

Polygenic risk scores (PRSs) have become an increasingly popular modality for the use of genetics in medicine, particularly for the early detection of individuals with high genetic risk for a given disease [15]. With the steady expansion of genome-wide association studies (GWASs), genetic data on multiple diseases are now readily available for PRS production. PRSs are developed through the use of GWAS data to determine the single nucleotide polymorphisms (SNPs) associated with the development of a given disease or the phenotype of interest. Specifically, the number of risk alleles at each locus is determined (either 0, 1, or 2) and then weighted by the associated β-value (a measure of relative risk of the effect allele vs. the reference allele) from the discovery GWAS. While the effect of one SNP is generally quite small, by using an additive model and summing the effects of all contributing SNPs in the DNA of an individual, a composite score can be produced and then compared to a reference population for relative genetic risk determination. This method has the potential to scale far more broadly across a population because it does not rely on single-gene testing and can be applied, at a relatively low cost, as a screening and disease prevention tool. Others have demonstrated a significant improvement to the development of PRS algorithms and the potential benefits of their use for categorizing individuals into specific risk categories [16]. By doing so, patients would be given more personalized advice on lifestyle choices, screening methods, and treatment options to prevent or manage a disease [16].

In a previously published study by Mavaddat et al. in 2019, the authors developed BC PRSs optimized for the prediction of estrogen receptor-specific BC, utilizing a large collaborative effort involving 94,075 BC positive cases and 75,017 BC negative controls of European ancestry genotyped on the iCOGS and OncoArray following 1000 genomes imputation [17]. The PRSs were independently and prospectively validated in a test dataset comprising 11,428 breast cancer-affected cases and 18,323 controls. The two best PRSs, 313-SNP and 3820-SNP PRSs, were developed using hard-thresholding, least absolute shrinkage, and selection operator (LASSO) methods, respectively. Validation testing revealed area under the receiver operating characteristic curve (AUROC) values of 0.639 and 0.646 for overall BC for the 313-SNP and 3820-SNP PRSs, respectively. When tested prospectively, the AUROC values decreased slightly to 0.630 and 0.636 for overall BC, respectively, for the 313-SNP and 3820-SNP PRSs. Though the AUROC values were modest, the differences in the tails of the distribution of predicted risk were large, as demonstrated by women in the top 1% of the distribution having a predicted risk approximately four-fold larger than the risk of those in the middle quintile. Furthermore, the association between PRS and disease risk was observed for women with and without a family history of disease.

The clinical potential of PRSs faces a significant hurdle related to the diverse genotyping methods used to procure the necessary genetic data. When essential variants crucial for PRS calculations are not directly identified by genotyping platforms, genotype imputation becomes essential. This process leverages high linkage disequilibrium (LD) genetic variants to infer untagged SNPs, reducing costs compared to whole-genome sequencing. While imputation has been successful, it becomes less precise with rare alleles, potentially yielding slightly different genotypes. These variations, especially at critical loci, may alter an individual’s overall genetic risk, impacting the resulting PRS and overall risk categorization [18]. Such variability raises concerns regarding proper risk stratification and subsequent clinical care. Here, we assess the variability in BC PRS due to the imputation process.

This study aims to replicate and assess the efficacy of two previously established PRSs within a cohort of BC cases and cancer-negative controls. Additionally, we aimed to assess the impact of genotype imputation on BC PRSs. We anticipate that these PRSs will effectively gauge BC risk among our study subjects, showing comparative performance to prior findings. Additionally, we expect slight variations in BC PRSs due to imputation disparities and the inherent probabilistic nature of genotype inference from reference populations. Through validation across two independent European ancestry populations, this research aims to bolster the evidence supporting PRSs’ utility in differentiating high- and low-risk individuals, facilitating improved stratification for screening and preventative therapies while optimizing risk–benefit ratios. Furthermore, our findings offer insights into the influence of genotype imputation on PRSs, presenting potential strategies for addressing associated concerns.

## 2. Materials and Methods

### 2.1. Study Subjects

**Figure 1:**
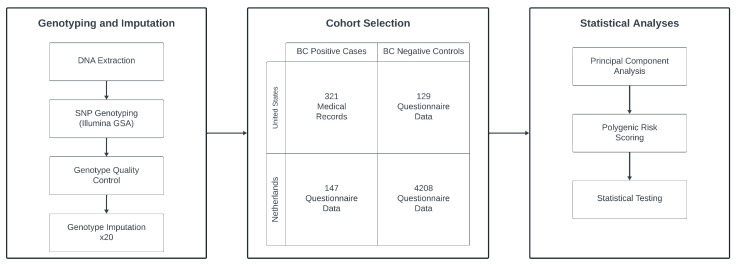
Diagram of the general study design. Ultimately, 468 cases and 4337 controls from the US and NL were selected after quality control. Genotyping was performed for all subjects, undergoing quality control steps, and 20 rounds of imputation. Data were then analyzed using principal component analysis, polygenic scoring, and various statistical tests to produce the study results.

We curated two European ancestry cohorts of breast cancer (BC) cases and controls originating from the United States (US) and the Netherlands (NL), as shown in Figure 1. The US-based BC-positive cases (*n* = 321) were sourced from the integrated Cancer Repository for Cancer Research (iCaRe2) and the Breast Cancer Collaborative Registry (BCCR), situated in the Midwestern United States [19]. Concurrently, the US BC-negative controls (*n* = 129) were drawn from the Avera Twin Register [20]. The NL cohort comprising 147 BC cases and 4208 controls, was obtained from the Netherlands Twin Register, part of the Vrije Universiteit in Amsterdam [21]. Previous quantitative analyses have confirmed the genetic similarity between these populations [22]. Participant inclusion criteria encompassed genetically confirmed female sex, European ancestry, and genetic non-relatedness.

### 2.2. Phenotypes

Phenotypic data for the US case subjects were gathered via IRB-approved patient questionnaire and electronic medical records using the iCare system. For the NL cases, phenotype information was obtained from longitudinal questionnaire data based on female self-reporting of breast cancer and the question, “Did you ever have cancer?” and the follow-up question “What type of cancer?”. The consistency between the questionnaire data and the Netherlands Cancer Institute medical record information (Netherlands Cancer Registration application K09.56) was checked in 2010, and at the time showed full concordance. Phenotype data for control subjects were collected via longitudinal questionnaires sent to the participants. Priority was given to the most recent available data for control ages and all records were searched to confirm cancer-negative status. All case and control subjects consented to the collection of biological specimens and phenotypic data according to Avera Internal Review Board approval or Central Ethics Committee on Research Involving Human Subjects of the Vrije University Medical Centre.

### 2.3. Genotyping and Imputation

A total of 20,723 subjects were genotyped on the Illumina Global Screening Array (GSA) according to the manufacturer’s protocol. Subjects had DNA extracted from blood or buccal samples. Blood samples were collected via a phlebotomist within the Avera hospital system in an 8 mL PAXgene DNA tube. Samples were then stored at −20 °C for long-term storage. DNA extraction for case and control subjects was either performed manually via the PAXgene DNA extraction kit or was performed using the Qiagen QiaSymphony platform and stored at −20 °C. Subjects were genotyped on the Illumina Global Screening Array (GSA) according to the manufacturer’s protocol. Genotype calling and quality control were conducted initially via GenomeStudio2.0 software. PLINKv1.9 [23] was used for additional quality control to confirm female biological sex of the DNA samples. For the NTR, the identity-by-descent status was compared to the expected relational status. Samples with a PLINK heterozygosity F-value less than −0.10 or greater than 0.10 were removed. Samples were retained if the call rate was above 90%, as well as above 80% per chromosome. All samples failing these criteria were excluded. Then only female samples with European ancestry and BRC phenotypes available were selected. KINGv2.2.5 [24] was used to estimate genetic relatedness. Up to 3rd degree relatives were finally excluded from subsequent analyses to avoid PRS value distribution differences due to different measures of relatedness in cases and controls. The total sample size after all exclusions was 4805, but only 12 cases were dropped. The number of samples removed in each step as well as the number of single nucleotide polymorphisms (SNPs) removed from the data are reported in Appendix A Table A1 and Table A2, respectively.

SNPs (*n* = 669,317) were checked and excluded based on the following criteria: duplicate SNPs and non-relevant mitochondrial and chromosome Y SNPs, >5% Mendelian inheritance problems in NTR families, genotyping error rate—defined by >5% genotype mismatches in 370—2 times genotyped—samples, call rate < 95%, Minor Allele Frequency (MAF) < 0.005, and Hardy–Weinberg Equilibrium (HWE) *p*-value < 0.0001. SNPs were also excluded if they were absent from the 1000 Genomes [25] V3 Phase 5 reference panel (1000 G), and if the allele frequency of the SNPs exceeded a 0.10 difference with the European reference subset in the 1000 G panel. Palindromic SNPs with allele frequencies between 0.40 and 0.60 were excluded and SNPs were removed if the alleles did not match after strand alignment, which happens if there are more than 2 SNP alleles. Genotype imputation to the 1000 G reference panel was performed using BEAGLE 5.4 (22jul22.46e) software [26] on the 518,924 SNPs that remained following quality control. The imputation process was repeated 20 times with different random seeds to obtain stochastic variability of inferred genotypes. The imputed genotype probabilities of each set were converted to best-guess genotypes with PLINK for roughly 31.6 million genetic variants.

### 2.4. Statistical Analyses

#### 2.4.1. Genetic Principal Component Analysis

Principal component analysis (PCA) was performed with PLINK using the 1000 G reference data for the African, East Asian, and European superpopulations combined with study population data. SNPs that passed SNP quality control in the study sample and that were also in the HapMap3 and the 1000 Genomes reference sets, with MAF > 0.01 and call rate > 98% were used for PCA. These SNPs were pruned for LD with a pairwise independence window of 1 MB and a maximum LD-R^2^ of 0.20. Long-range linkage disequilibrium regions were excluded [27] leaving a total of 69,717 SNPs. Only individuals with European ancestry were selected which were identified by having PCs within the maximum and minimum values of the first 10 PCs of the 1000 G European superpopulation. To avoid residual population stratification, PCs were also used as covariates in downstream analyses.

#### 2.4.2. Polygenic Risk Scoring

Summary statistics for both the 313-SNP and 3820-SNP sets, containing the effect estimates, SNP names, and risk alleles needed for polygenic scoring, were downloaded from the supplementary materials published as part of the Mavaddat et al. study [17]. These were matched with the imputed study genetic data to make sure that the names, and risk and alternate alleles of each SNP were equal to our study data. This led to a minor reduction in the number of SNPs due to there being more than two mismatching alleles present in a SNP and unresolvable strand designation (for A/T and C/G SNPs) between the Mavaddat sets and our study data. When possible, bi-allelic alternative tagging SNPs were used to replace these alleles. This was achieved by uploading a list of the affected SNPs to the Ensembl database (build 37) and then using a combination of two measurements of linkage disequilibrium between SNPs, namely a maximal D’ and LD-R^2^, a minimal allele frequency difference for alleles, and a minimal distance to the original SNP, as subsequent selection criteria for determining the best alternative SNP present in our data. This resulted in retaining 307 out of the 313 (98.1%) and 3712 out of the 3820 (97.2%) SNPs. Polygenic scores were then calculated for all individuals in our study using the PLINK v1.90 software for all 20 best-guess imputation replicates.

For generating the percentile scores, the minimum and maximum polygenic scores were taken from each replicate run. Subsequently, for each individual a percentile score was calculated by taking the maximum minus the minimum individual score, divided by the range (maximum–minimum) and multiplying by 100%. Over the 20 replicate imputations, the average percentile score was then calculated, as well as the minimum and the maximum percentile score for each subject.

#### 2.4.3. Statistical Testing

Statistical testing was performed in either SPSSv28 or Python v3.12. For assessing mean differences between cases and controls, the 20-run average percentile polygenic score was used in a one-way ANCOVA. Age and was used as a covariate for all means testing. A two-stepwise logistic regression was used to determine the explained variance by the Nagelkerke R^2^, a measure of goodness-of-fit, for the PRS. In the first step, case control status was predicted by the 10 PCs plus age. In the second step, either the 313 PRS or the 3820 PRS or both were added to the model to determine the improvement in the Nagelkerke R^2^ to test how well each PRS predicted and which PRS set predicted the best. PRS prediction was also determined with AUROC calculations based on the 20-run average PRS percentile for cases and controls from all 20 imputation runs. A Pearson correlation was employed between the average 313 and 3820 PRSs in the whole study sample to examine the equality of the two scores. Absolute risk measures were calculated with the GenoPred (https://opain.github.io/GenoPred/PRS_to_Abs_tool.html) interactive webtool provided by Pain et al. [28] by inputting population standardized PRS, population-specific AUC, and a population-specific BC disease prevalence estimate. Figures and tables were generated using a combination of Microsoft Excel v2401, SPSS v28, and Python v3.12. Since a significant difference between the polygenic scores was found between the NL and US cases, we performed an χ^2^—association test comparing the allele frequencies of the 307 and 3712 SNPs between both cases as well as controls, to confirm absence of an allele difference here. The α-level of these tests that we considered significant was *p* < 0.001.

## 3. Results

### 3.1. Population Characteristics

**Figure 2:**
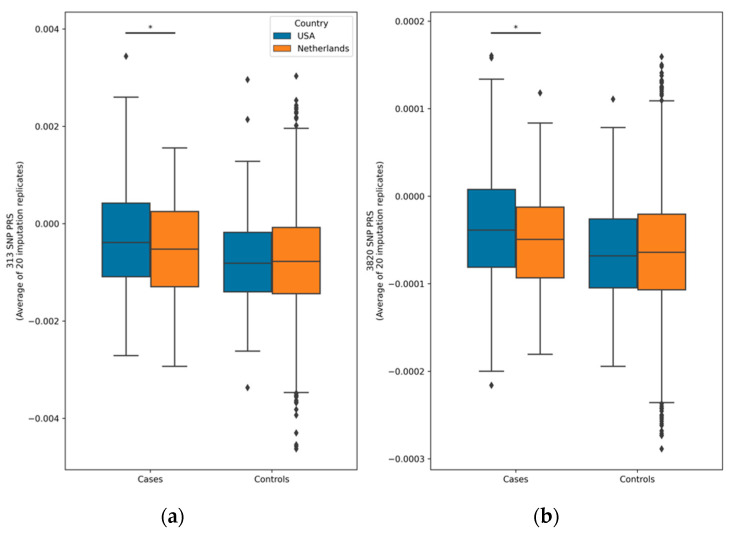
Box plots of polygenic risk score distributions. The asterisks (*) represent statistical significance levels (*p* < 0.05) from an ANCOVA test comparing cases and controls within the Netherlands and the USA for both the 313-SNP (**a**) and 3820-SNP (**b**) PRS models. Notably, cases and controls from each country showed significant differences (*p* < 0.01) for both PRS models, although the significance bars are not depicted. Each PRS value presented reflects the average across all 20 imputation replicates. Diamonds indicate PRS value outliers.

Table 1 contains the population characteristics and the average percentile score over the 20 imputation replications, separately for the American (USA) and Dutch populations (NL). Note that not all 313 and 3820 SNPs could be utilized, resulting in the retention of 307 and 3712 SNPs, respectively. The PRS distributions for cases and controls separated by country are shown in Figure 2. For both the 313-SNP and 3820-SNP PRS models within each country, there was a significant difference between cases and controls (*p* < 0.01 for all comparisons). The overlap between the cases and controls can be seen in the scores and is common in PRS studies given the probabilistic nature of genetic risk. There was also a significant difference between American and Dutch cases for both PRS models.

### 3.2. Comparison of PRS Prediction Performance in BC Cases and Controls

Using a stepwise logistic regression with 10 PCs to account for residual population stratification, age of onset for cases, and age of last report of not having been affected by BC for controls as covariates, we examined the effect of the average 313-SNP and 3820-SNP PRSs in predicting BC disease status in the total study population. The results of the modeling showed a significant effect of prediction of the 313-SNP PRS indicated by the significant regression coefficients (beta = 0.030, SE = 0.004, *p*-value = 1.9448 × 10^−13^) and of the 3820-SNP PRS (beta = 0.029, SE = 0.004, *p*-value = 3.402 × 10^−15^). The addition of the 313-SNP PRS to the model increased the Nagelkerke R^2^ from 0.127 to 0.15. The addition of the 3820-SNP PRS to the model increased the Nagelkerke R^2^ from 0.127 to 0.153.

**Figure A1:**
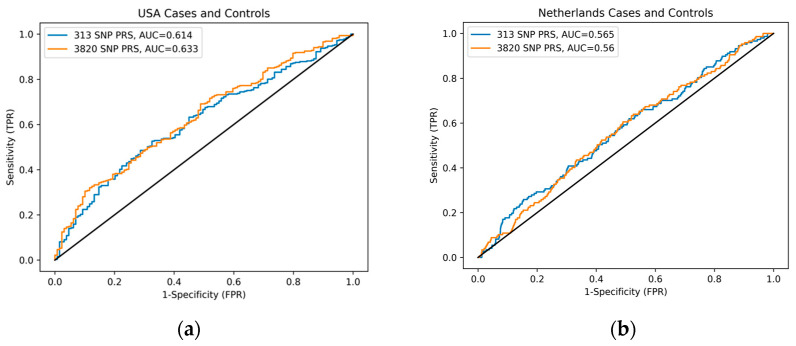
ROC curve comparing 313-SNP and 3820-SNP PRS performance by country. (**a**) ROC curve of US cases and controls with an AUC of 0.614 and 0.633 for the 313-SNP and 3820-SNP PRS, respectively. (**b**) ROC curve of NL cases and controls with an AUC of 0.565 and 0.560 for the 313-SNP and 3820-SNP PRS, respectively.

We also assessed the same models in each country separately (Figure A1). In the NL population, using the 313-SNP PRS, the logistic model showed an effect on the prediction of BC (beta = 0.021, SE = 0.007, *p*-value = 0.002) with an Nagelkerke R^2^ increase from 0.069 to 0.077. For the 3820-SNP PRS, the values (beta = 0.018, SE = 0.006, *p*-value = 0.0026) for R^2^ also increased from 0.069 to 0.077. In the American samples, the logistic regression model using the 313-SNP PRS showed a slightly larger predictive value (beta = 0.024, SE = 0.009, *p*-value = 0.011). There was an even more predictive value for the 3820-SNP PRS (beta = 0.029, SE = 0.009, *p*-value = 0.0015). For both the 313-SNP and 3820-SNP PRS, the Nagelkerke R^2^ of the model increased from 0.293 to 0.311 and 0.320, respectively. A logistic forward conditional stepwise model corrected for country showed that the 3820-SNP PRS predicts better than the 313 PRS.

**Figure 3:**
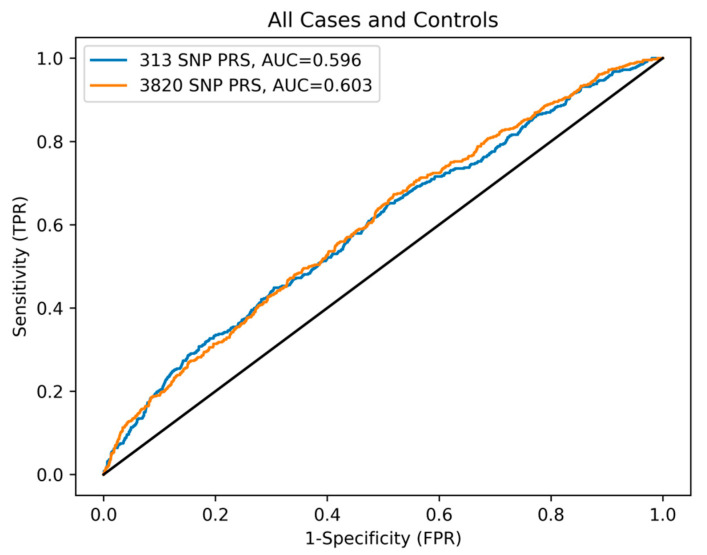
ROC curve comparing 313- and 3820-SNP PRSs performance. The ROC reference is indicated by the solid black line.

To best compare the 313-SNP and 3820-SNP PRSs to each other, as well as the paper by Mavaddat et al. [17], we utilized the area under the curve (AUC) receiver operator characteristic (ROC) to compare the general performance of the PRSs across all possible thresholds. Here, the PRSs were used as a predictor for disease state. For the US data, the AUC values were 0.614 (95% CI 0.558–0.669) and 0.633 (95% CI 0.578–0.688), for the 313-SNP and 3820-SNP PRSs, respectively. While for the NL data, the AUC values were 0.565 (95% CI 0.517–0.612) and 0.560 (95% CI 0.514–0.607) (see Figure A1). As shown in Figure 3, the combined data had AUC values of 0.596 (95% CI 0.569–0.624) and 0.603 (95% CI 0.576–0.630), respectively. These results indicate a moderate increase in predictive performance for the 3820-SNP PRS in each population and for both PRSs in the US data compared to the NL data.

### 3.3. Assessment of Imputation Replication on PRS Variability

In this study, we used 20 imputation replications to get an average PRS score for each person. Here we examine the variability of the percentile PRSs within each individual. We calculated the minimum and maximum PRS percentile of each person and then subtracted the two to get a range of score variability. The range of PRS variability was then averaged over all persons. The average percentile PRS score variability over all individuals (*n* = 4805) is 2.22% (SD 0.90) for the 313-SNP PRS and 3.03% (SD 0.84) for the 3820-SNP PRS. In the various subgroups analyzed, the variability is as follows for the 313-SNP PRS: US cases 2.52% (SD 1.04), US controls 2.64% (SD 0.94), NL cases 2.23% (SD 0.99) and NL controls 2.18% (SD 0.88). Likewise for the 3820-SNP PRS: US cases 3.42% (SD 1.03), US controls 3.36% (SD 0.80), NL cases 2.97% (SD 0.87) and NL controls 2.98% (SD 0.81). Both PRSs show a slightly larger variation in the American scores compared to the Dutch (*p* < 0.004), in both cases and controls. There were no significant differences in score variability between cases and controls within each country (*p* > 0.252). The largest difference between the minimum and maximum imputed scores for any individual in the study population for the 313-SNP PRS is 8.56%, and 9.15% for the 3820-SNP PRS. The Pearson correlation between the 313-SNP and 3820-SNP PRSs is strong at 0.825, but not perfect unity.

Given the increased predictive power and the overall higher scores in the US samples, we also examined the SNP markers resulting in this variation. Following imputation there were seven variants which show a substantial allele frequency difference (defined by an association *p*-value within cases *p* < 0.001) between the American and Dutch cases. The resulting markers and their allele frequencies are shown in Table 2 as well as the expected effects based on the 3820-SNP PRS summary statistics.

### 3.4. Relative and Absolute Risk Estimation

To aid in the interpretation of BC risk, we used the GenoPred interactive webtool provided by Pain et al. [28] to convert the American and Dutch case and control group PRSs to the absolute scale for each PRS model. To calculate absolute risk, we first standardized the PRS within each country subpopulation. Utilizing mean case and control PRS Z-scores for each population, the corresponding population-specific AUC of each PRS, and a BC prevalence estimate of 2.8% for non-Hispanic females in the United States [29], we calculated relative and absolute risk for the 313-SNP and 3820-SNP PRSs. The results of are shown in Table 3. For example, assuming a population BC prevalence of 2.8% and a polygenic AUC of 0.614 for the 313-SNP PRS for American cases, the average American case has a polygenic Z-score of 0.11, they are in the 54.5th percentile of BC PRS. Converting to the absolute scale reveals that 2.60% of individuals with that PRS will develop BC, based on the PRS Z-score and corresponding AUC of the subpopulation PRS. Comparatively, the average American control had a polygenic Z-score of −0.276. Using the same polygenic AUC of 0.614 for American samples, they would be in the 39.4th percentile of BC PRS. Conversion to the absolute scale again suggests that 2.20% of individuals with that PRS will develop BC, indicating a lower overall absolute risk than the American cases for the 313-SNP PRS.

We also conducted a thorough evaluation of both relative and absolute risk estimation stratified by PRS percentile bins (0–25%, 25–50%, 50–75%, and >75%), aiming to provide a nuanced understanding of risk estimates grounded in calculated genetic predisposition in both the US and NL cohorts (Table A3). Our analysis reveals a consistent trend wherein relative and absolute risk estimates rise with PRS percentile, with case risks generally exceeding those of controls across both populations. Nevertheless, certain deviations from this pattern exist, primarily attributable to constrained and unequal sample sizes, as well as the influence of disease prevalence and AUC values on estimates. It is essential to acknowledge that controls were defined based on their current state, which may evolve to include individuals who develop the disease over time.

## 4. Discussion

Significant progress in genotyping technology has allowed for the accrual of large amounts of genetic data around the world. Over the last decade, researchers have developed methods for making PRSs for various diseases which have the potential to accurately determine the genetic risk of an individual for a given disease and to help improve disease prevention efforts. In this study, our goal was to validate the use of two PRSs for BC in representative case and control populations and to examine the effect of genotype imputation on PRS. Overall, our investigation into PRS models for BC prediction revealed intriguing insights, shedding light on the nuanced interplay between genetic predisposition, population characteristics, and predictive accuracy across different cohorts of similar genetic ancestry.

Based on our analyses, we can confirm that both the 313-SNP and 3820-SNP PRSs perform moderately well in distinguishing individuals with and without BC. We used PCA to generate standardized residual scores for reliable comparisons and used age as a covariate to ensure that the limited allele frequency differences in the case and control cohorts were corrected for and to account for potential confounding. Our data showed a significant difference between the case and control groups for both the 313-SNP and 3820-SNP PRS models (*p* < 0.01), with the case mean PRSs being significantly greater than the mean PRSs of the respective control groups, confirming the capacity for the 313-SNP and 3820-SNP PRS models to distinguish BC cases from controls.

Our analysis of the 313-SNP and 3820-SNP PRS models further delineated their performance across populations. Notably, both PRS models exhibited significant predictive power for BC, with the 3820-SNP PRS demonstrating superior performance in both study cohorts. This observation was consistent across the overall study population and within individual countries.

The logistic regression models emphasized the substantial enhancement in Nagelkerke R^2^ values upon inclusion of both PRS models, underlining their utility in BC prediction. Additionally, the area under the curve (AUC) values derived from receiver operator characteristic (ROC) analysis showcased moderate but consistent improvements in predictive performance for the 3820-SNP PRS compared to the 313-SNP PRS, across both American and Dutch populations.

A noteworthy finding was the disparity in prediction accuracy between the American and Dutch cohorts. Our study indicated a higher predictive value of PRS for BC in the American samples compared to those obtained from the Netherlands. We highlight potential factors that may explain this discrepancy. Firstly, the genetic proximity of the American samples to the original GWAS population and study by Mavaddat et al. [17] potentially conferred a higher predictive accuracy. Secondly, we identified seven variants exhibiting significant allele frequency differences between the Dutch and American samples, which unveiled a plausible reason for the divergent predictive abilities in the two populations. These variants possessed the largest allele frequency differences between cases from the two populations and potentially underpin the enhanced predictive value observed in the American population. The identified variants warrant further investigation to elucidate their functional relevance in breast cancer susceptibility, potentially enriching the predictive power of future PRS models for specific populations. Finally, the distinct nature of case identification—diagnosed breast cancer cases in the USA based on medical records versus population-based surveys in the Netherlands—might contribute to the varying severity levels captured within the cohorts, potentially impacting the overall predictive accuracy of the PRS models.

Moreover, our investigation into the impact of genotype imputation on BC PRSs revealed noteworthy findings. Through 20 imputation replications, we observed a 2–3% variation in resultant BC PRS, aligning with previous findings [18]. The study demonstrated that utilizing different pre-phasing and imputation tools resulted in minimal percentile changes (<5%) across 14 PRSs, encompassing different disease architectures and PRS calculation approaches. However, in our study, there were also people with substantially larger differences, even reaching up to 9%. This highlights the challenges in individual-level genetic analysis where rare variability events can be obscured by high overall score reproducibility at the population level. To address potential fluctuations in the PRS results of some individuals, our suggestion echoes that of Chen et al. [18], in that our recommendation would be to employ the average of multiple imputation iterations when calculating PRSs for clinical use and or personal predictions, when using imputed genotype data. This will mitigate the stochastic nature of inferring alleles, ensuring more robust and consistent PRS outcomes.

Understanding how PRS assesses risk and how this may impact clinical decision making, it is important to differentiate between relative and absolute risk. To do so, we utilized the GenoPred web tool [29] to convert PRS to relative risk and absolute risk for each of our case and control populations. The relative risk differences were approximately 10–15% between cases and controls for the 313-SNP PRS in both the US and NL cohorts. This relative risk difference expanded in the US cohort to nearly 20% and shrank to about 8% in the NL cohort for the 3820-SNP PRS. The absolute risk for the US cohort was 0.40% greater for the average US case (2.60%) vs. the average US control (2.20%) based on the 313-SNP PRS. Compared to the absolute risk reduction (ARR) of 20 years of screening mammograms (0.49%) [30], the ARR for the 313-SNP PRS of 0.40% is smaller, but not without impact, especially given that the test would only need to be performed once and at an earlier age than mammography. PRS testing may, in fact, be able to improve patient selection for increased mammography frequency or use at an earlier age, as some studies suggest [31].

This ARR is increased as the PRS increases, as exemplified by a US BC case in our study with a Z-score of 1, the absolute risk is 3.7% (based on 313-SNP PRS AUC), leading to an absolute risk reduction of 1.5% compared to the average US control. As the PRS further increases to a Z-score of 2, the absolute risk becomes 5.50% with a resulting absolute risk reduction of 3.30% compared to the average US control. These calculations suggest that PRS functions to distinguish individuals at the higher end of genetic risk better than individuals closer to the mean. However, these comparisons are difficult to truly assess because PRS functions as a spectrum, rather than a yes–no screening test, leading to lost context.

The results of the percentile bin RR and AR calculations show much smaller differences when comparing similar quartiles between cases and controls, especially for the NL population, where the ARR is often 0.1%. In addition, the combination of our smaller sample size, relatively low disease prevalence, and low AUC likely contributes to these results. Larger prospective studies will likely improve the field’s ability to address these questions.

While our study yields valuable insights into BC PRS modeling, acknowledging limitations is crucial. It is imperative to note that our PRS analyses were conducted after excluding certain SNPs from the 313-SNP and 3820-SNP PRSs. Whenever feasible, direct bi-allelic tagging SNPs were used as replacements, but nevertheless resulted in PRS calculations based on 307 out of the 313 (98.1%) and 3712 out of the 3820 (97.2%) SNPs initially considered. Additionally, the use of population-based surveys for cases in the Netherlands and the potential influences of unaccounted genetic or environmental factors may have contributed to the diminished predictive performance compared to the clinically diagnosed BC cases from the US. The data collected in this study did not contain sufficient information on all cases regarding cancer stage or histochemical subtype, limiting our ability to investigate these characteristics in our study population. This discrepancy highlights the need for ongoing research to refine and enhance the accuracy of PRS models tailored to specific subtypes of disease and diverse populations more generally. Additionally, it is vital to recognize that PRSs are not entirely specific to predicting only the intended trait. They may also forecast other pertinent disease comorbidities or genetically correlated traits. For instance, a documented positive genetic correlation exists between BC and schizophrenia (rg  =  0.14, se  =  0.03) [32]. Therefore, variations in PRS predictive performance could stem from prevalence differences of genetically correlated traits among populations.

## 5. Conclusions

In conclusion, our study underscores the promising potential of PRS in BC risk assessment, emphasizing the importance of refining models to suit diverse populations and addressing variations observed across cohorts for more accurate predictive outcomes.

## Figures and Tables

**Table 1:** Average age and PRS of study population.

	USA		Netherlands		Total (USA and Netherlands)	
	Controls	Cases	Controls	Cases	Controls	**Cases**
**Sample Size**	129	321	4208	147	4337	468
**Average Age**	42.60 (15.21)	57.19 (12.14)	45.34 (16.84)	56.86 (9.76)	45.25 (16.80)	57.08 (11.43)
**313-SNP PRS Avg. %**	48.51 (12.00)	53.66 (13.56)	48.06 (12.63)	50.95 (12.11)	48.07 (12.61)	52.81 (13.17)
**3820-SNP PRS Avg. %**	49.64 (12.94)	56.61 (14.46)	49.81 (14.16)	52.94 (13.47)	49.81 (14.32)	55.45 (14.32)

All subjects were genetically confirmed to be female, of European ancestry, and unrelated. Standard deviation values listed in parentheses where appropriate.

**Table 2:** SNPs exhibiting allele frequency differences between US and NL cases and controls.

					US Allele Frequency		NL Allele Frequency			Summary Statistics	
rsID	Chr	BP	A1	A2	Cases	Controls	Cases	Controls	R^2^ *	Effect Allele	Effect Size
rs13291323	9	6185360	C	T	0.032	0.058	0.087	0.061	0.920	C	0.0046
rs7113140	11	123053078	T	C	0.488	0.434	0.371	0.428	1.000	T	0.0019
rs12296461	12	116347863	A	G	0.542	0.552	0.655	0.553	0.970	A	0.0048
rs12907670	15	63742901	G	A	0.832	0.857	0.913	0.869	0.980	G	0.0102
rs4984247	15	63758647	C	T	0.850	0.892	0.926	0.884	0.970	C	0.0044
rs34853502	16	53865368	A	AG	0.319	0.279	0.216	0.258	0.990	A	0.0145
rs13049602	21	33501003	C	T	0.826	0.810	0.731	0.780	0.961	C	0.0082

* Average Beagle imputation quality R^2^ (0–1) over 20 genotype imputation runs.

**Table 3:** Relative and absolute risk calculations at observed mean PRS Z-scores.

		US		NL	
SNP Panel		Cases	Controls	Cases	Controls
**313-SNP PRS**	Relative Risk distribution of PRS	54.40%	39.40%	59.10%	49.60%
	Absolute Risk distribution of PRS	2.60%	2.20%	2.80%	2.60%
**3820-SNP PRS**	Relative Risk distribution of PRS	55.20%	36.70%	58.30%	49.60%
	Absolute Risk distribution of PRS	2.60%	2.10%	2.80%	2.60%

## Data Availability

Restrictions apply to the availability of these data. Data were obtained from the integrated Cancer Repository for Cancer Research (iCaRe2), Netherlands Twin Register (NTR), and the Avera Twin Register (ATR) and are available from the authors with the permission of the iCaRe2, NTR, and ATR. For the NTR, a data request can be performed at the following webpage: https://ntr-data-request.psy.vu.nl/ (accessed 15 January 2023).

## Appendix A

**Table A1:** Samples removed per study quality control procedures.

Sample QC	Samples (n)
All subjects with genotyped on GSA (US + NL)	20,723
Callrate < 90%	−224
Multiple DNA measurements same person	−384
Heterozygosity	−208
Sex problems	−310
IBD problems	−172
	19,425
Sample filters	
Select only female members	11,580
Remove Non-European samples defined by 1000 Genomes PC projection.	11,225
Select women with a valid case control status	6997
20 × 1000 genomes imputation for 6997 individuals with random seed 1 to 20	
Selected sample for analysis (unrelated people only, keeping one of multiple related)	4805 *

***** Subpopulation breakdown in Figure 1.

**Table A2:** SNPs removed per study quality control procedures.

Genotyping QC Steps	SNPs (n)
Illumina GSA SNPs	669,317
Poor quality X Chr clustering	−271
Parametric Y Chr	−6
Duplicate markers	−1246
Absent allele markers (A/C/T/G)	−3503
Total	664,291
**SNP QC**	
Call rate < 95%	−5531
HWE < 0.001	−6688
MAF < 0.005	−130,095
Mendel errors ≥ 5%	−82
370 duplicate samples more than 5% genotype difference in SNP	−152
Palindromic, >2 alleles, allele frequency difference over 0.10, and removal of MIT + chrY	−2819
Total (pre-imputation and aligned)	518,924

**Table A3:** Relative and Absolute Risk Calculations on PRS percentile bins by country.

**US Cases**	**Bin**	**Subjects (n)**	**AVG Z-Score**	**RR (%)**	**AR (%)**	**US Controls**	**Bin**	**Subjects (n)**	**AVG Z-Score**	**RR (%)**	**AR (%)**
**313-PRS**	**≤25%**	2	−1.91	2.8	1.2	**313-PRS**	**≤25%**	1	−2.56	0.5	0.9
	**25–50%**	131	−0.57	28.4	2.1		**25–50%**	72	−0.61	27.1	2.0
	**50–75%**	165	0.90	81.6	3.7		**50–75%**	54	0.74	77	3.5
	**>75%**	23	2.58	99.5	7.2		**>75%**	2	3.18	99.9	8.3
**US Cases**	**Bin**	**Subjects (n)**	**AVG Z-score**	**RR (%)**	**AR (%)**	**US Controls**	**Bin**	**Subjects (n)**	**AVG Z-score**	**RR (%)**	**AR (%)**
**3820-PRS**	**≤25%**	2	−2.27	1.2	0.9	**3820-PRS**	**≤25%**	6	−1.92	2.7	1.0
	**25–50%**	106	−0.60	27.4	1.9		**25–50%**	60	−0.64	26.1	1.9
	**50–75%**	173	0.70	75.5	3.5		**50–75%**	60	0.60	72.6	3.3
	**>75%**	40	2.22	98.6	7.0		**>75%**	3	2.34	99	7.4
**NL Cases**	**Bin**	**Subjects (n)**	**AVG Z-score**	**RR (%)**	**AR (%)**	**NL Controls**	**Bin**	**Subjects (n)**	**AVG Z-score**	**RR (%)**	**AR (%)**
**313-PRS**	**≤25%**	3	−2.03	2.1	1.7	**313-PRS**	**≤25%**	142	−2.28	2.51	1.6
	**25–50%**	68	−0.52	30.2	2.4		**25–50%**	2220	−0.61	27.1	2.4
	**50–75%**	73	0.93	82.4	3.4		**50–75%**	1776	0.82	79.4	3.3
	**>75%**	3	2.25	98.8	4.5		**>75%**	70	2.51	99.4	4.8
**NL Cases**	**Bin**	**Subjects (n)**	**AVG Z-score**	**RR (%)**	**AR (%)**	**NL Controls**	**Bin**	**Subjects (n)**	**AVG Z-score**	**RR (%)**	**AR (%)**
**3820-PRS**	**≤25%**	2	−1.82	3.4	1.9	**3820-PRS**	**≤25%**	169	−2.19	1.4	1.7
	**25–50%**	56	−0.69	24.5	2.4		**25–50%**	1939	−0.68	24.8	2.4
	**50–75%**	80	0.67	74.9	3.2		**50–75%**	1961	0.69	75.5	3.2
	**>75%**	9	2.17	98.5	4.3		**>75%**	139	2.24	98.7	4.4
